# Supplementary material for: Conformational rigidity of cytochrome c'-α from a thermophile is associated with slow NO binding
Source: Biophys J. 2024 Jun 26;123(16):2594–603. doi: 10.1016/j.bpj.2024.06.026 (PMC11365222; doi:10.1016/j.bpj.2024.06.026)
Supplement: Document S1. Figures S1–S6 [file mmc1.pdf]

Biophysical Journal, Volume *123*

Supplemental information

Conformational rigidity of cytochrome *c*'-α from a thermophile is associated with slow NO binding

Sotaro Fujii, Michael T. Wilson, Hannah R. Adams, Halina Mikolajek, Dimitri A. Svistunenko, Peter Smyth, Colin R. Andrew, Yoshihiro Sambongi, and Michael A. Hough

Supplementary data

Conformational rigidity of thermophilic cytochrome *c*'-α associates with slow binding of nitric oxide

Sotaro Fujii,[a,b,c]* Michael T. Wilson,[d] Hannah R. Adams,[d] Halina Mikolajek,[a,b] Dimitri A. Svistunenko,[d] Peter Smyth,[a,b,d] Colin R. Andrew,[e] Yoshihiro Sambongi,[c,f] Michael A. Hough,[a,b,d]

[a]Diamond Light Source Ltd., Harwell Science and Innovation Campus, Didcot, UK

[b]Research Complex at Harwell, Harwell Science and Innovation Campus, Didcot, UK

[c]Graduate School of Integrated Sciences for Life, Hiroshima University, Higashi-Hiroshima, Japan

[d]School of Life Sciences, University of Essex, Wivenhoe Park, Colchester, UK

[e]Department of Chemistry and Biochemistry, Eastern Oregon University, La Grande, Oregon 97850, USA

[f]Seto Inland Sea Carbon-neutral Research Center, Hiroshima University, Higashi-Hiroshima, Japan

A

```
PhCP 1   DALKPEDKVKFRQASYTTMAWNMGKIKAMVVDGTMPFSQTQVSAAANVIAAIANSGMGALYSPDTLGVV 69
AxCP 1   QFAKPEDAVKYRQSALTLMASHFGRMTPV-VKGQAPYDAAQIKANVEVLKTLSALPWAA-FGPGT-E-G 65
SfCP 1   NFEEPADAIEYRQAAFGLIAYNFGDM-GAMLKGKKPFDAAVFSTRADNVAALSKIPHEG-FIAGS-DKG 66
             * *   **                  *

PhCP 70 GFKKSRLKENFFQEQDEVRKIATNFVEQANKLAEVAAMGDKDEIKAQFGEVGKACKACHEKFREEE 135
AxCP 66 G--DAR--PEIWSDAASFKQKQQAFQDNIVKLSAAADAGDLDKLRAAFGDVGASCKACHDAYRKKK 127 32%
SfCP 67 D-TEAL--AKIWQDKADFDSKMTAFQDNAAALAVAAKSSDQNNIKQAFANTGKSCKGCHDVYKKD- 128 27%
                         *      *   *   *        *  ** **
```

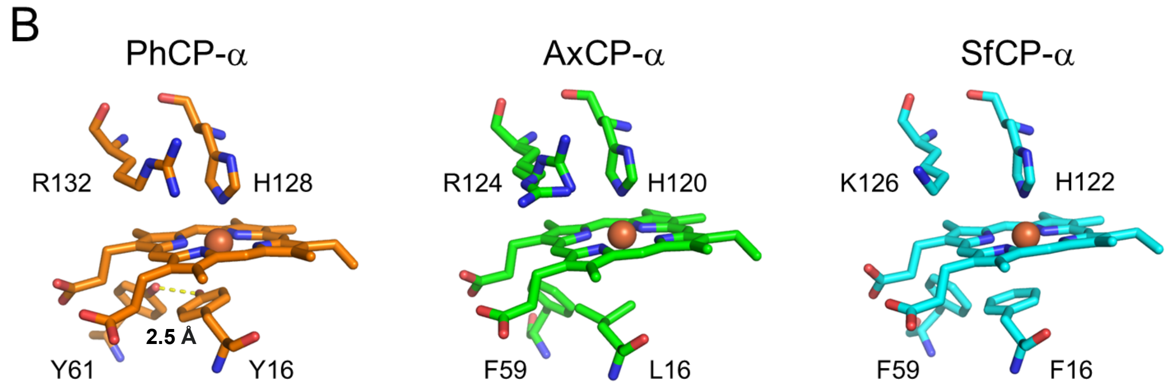

Figure S1

Structure comparison among cytochromes *c*'-α. (A) Amino acid sequence alignment of PhCP-α, AxCP-α, and SfCP-α. The relevant residues around the distal and proximal heme regions are shown in boxes. Asterisks indicate the identical residues among the three proteins. (B) Three-dimensional structure comparison of distal and proximal heme regions among PhCP-α, AxCP-α, and SfCP-α (PDB codes; 5B3I, 2YLI, and 4ULV, respectively). Boxed residues in panel A are shown. Yellow dotted line in PhCP-α indicates a hydrogen bond with a length of 2.5 Å.

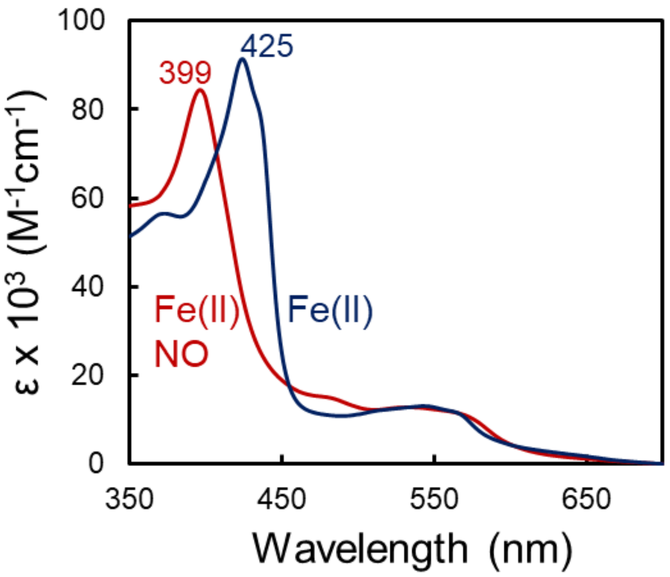

Figure S2

Molar extinction coefficient of UV-visible spectra of Fe(II) PhCP-α with NO (red line) or without NO (blue line).

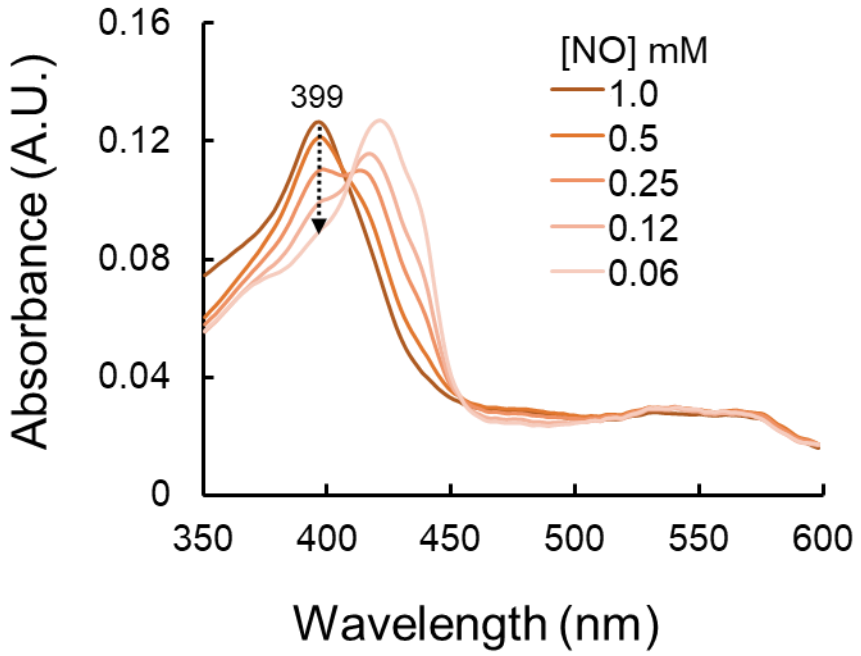

Figure S3

Absorption spectra of 2.5 µM Fe(II) PhCP-α with NO at pH 7.5 and 20 ºC after 5.5 seconds in stopped-flow analysis using white light with detecting the photodiode array.

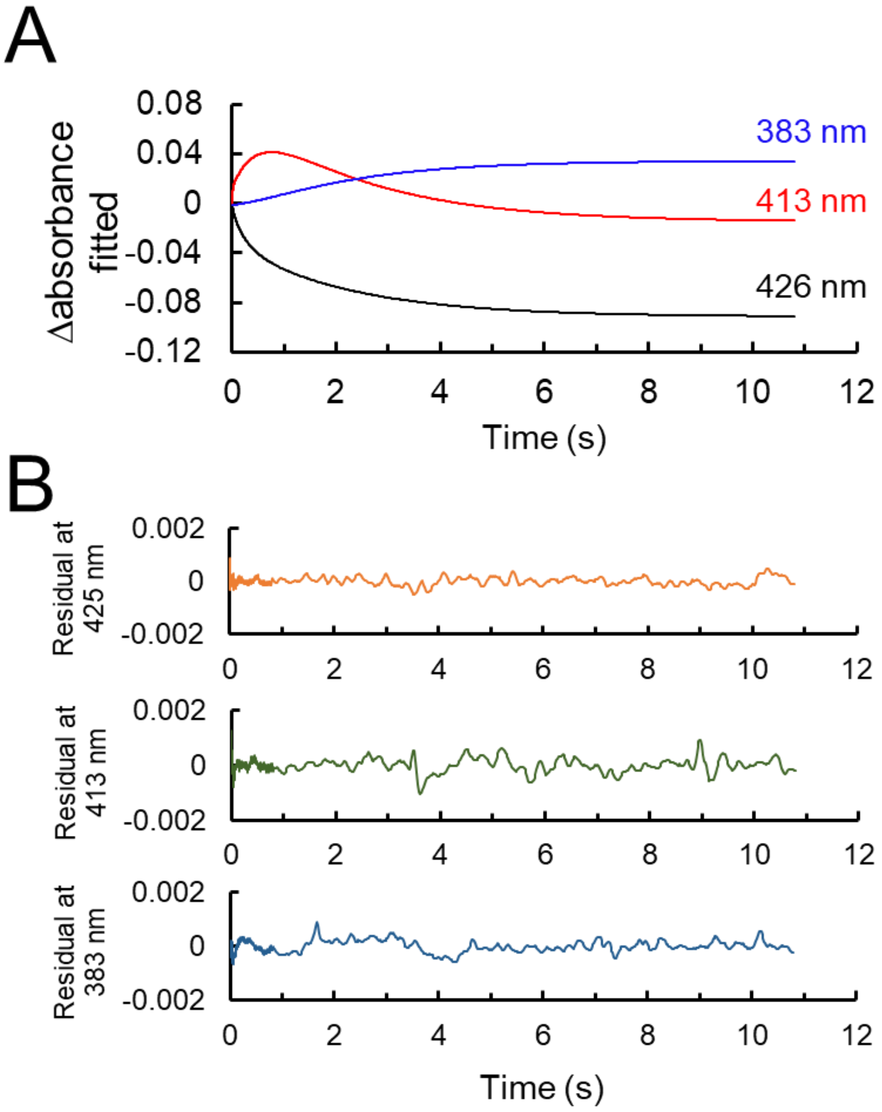

Figure S4

Stopped-flow kinetic analysis for PhCP-α using monochromatic light. (A) Fitted lines calculated from the time-dependent absorbance shift at 383 (blue line), 413 (red line), and 426 nm (black line) of the reaction of 2.5 μM Fe(II) PhCP-α and 1.0 mM NO at pH 7.5 and 20 °C. The absorbance at the start of 0.003 second is calculated as zero. (B) The residuals between raw data shown in Figure 3D and the corresponding fitted lines.

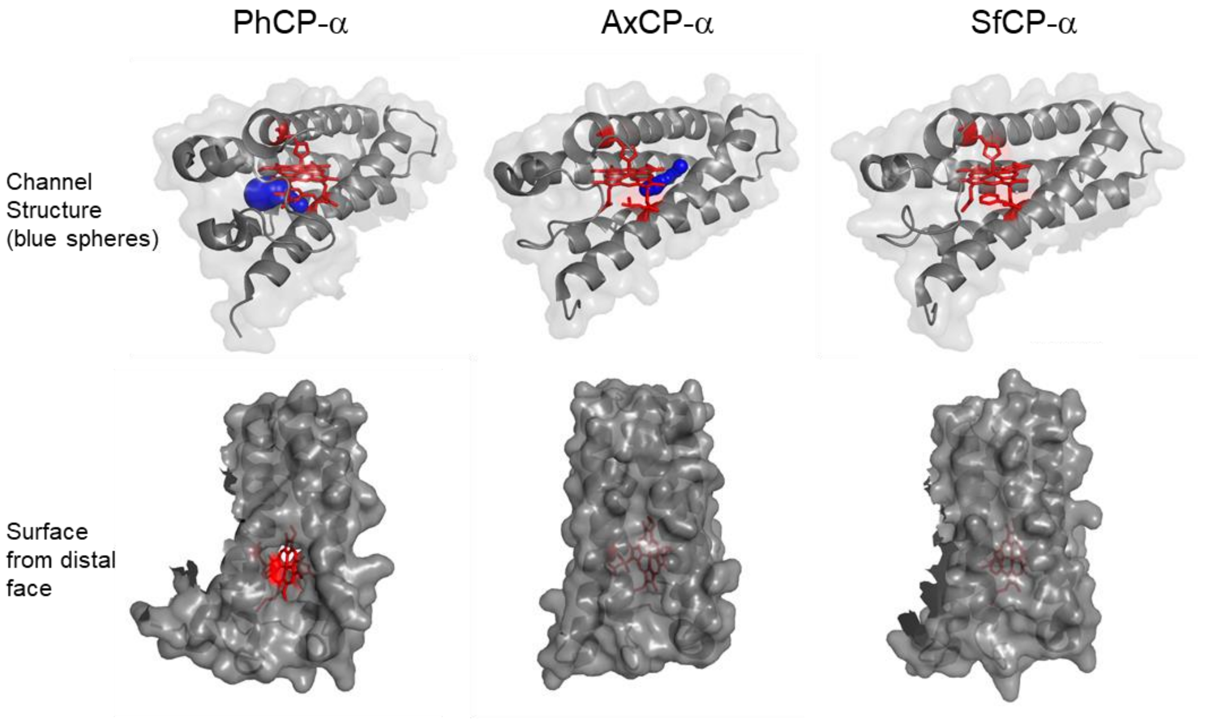

Figure S5

Channel structure analysis by CAVER program as PyMOL plugin (the top structures). The native structures of PhCP-α, AxCP-α, and SfCP-α (PDB IDs: 5B3I, 2YLI, and 4ULV, respectively) were used for the analysis. Blue spheres indicate the channel to the distal heme faces. The hemes with the coordinating distal and proximal His residues were shown by red sticks. The bottom structures indicate the protein surface shown in gray clouds, and only PhCP-α has a huge channel structure from the solvent to the distal heme face.

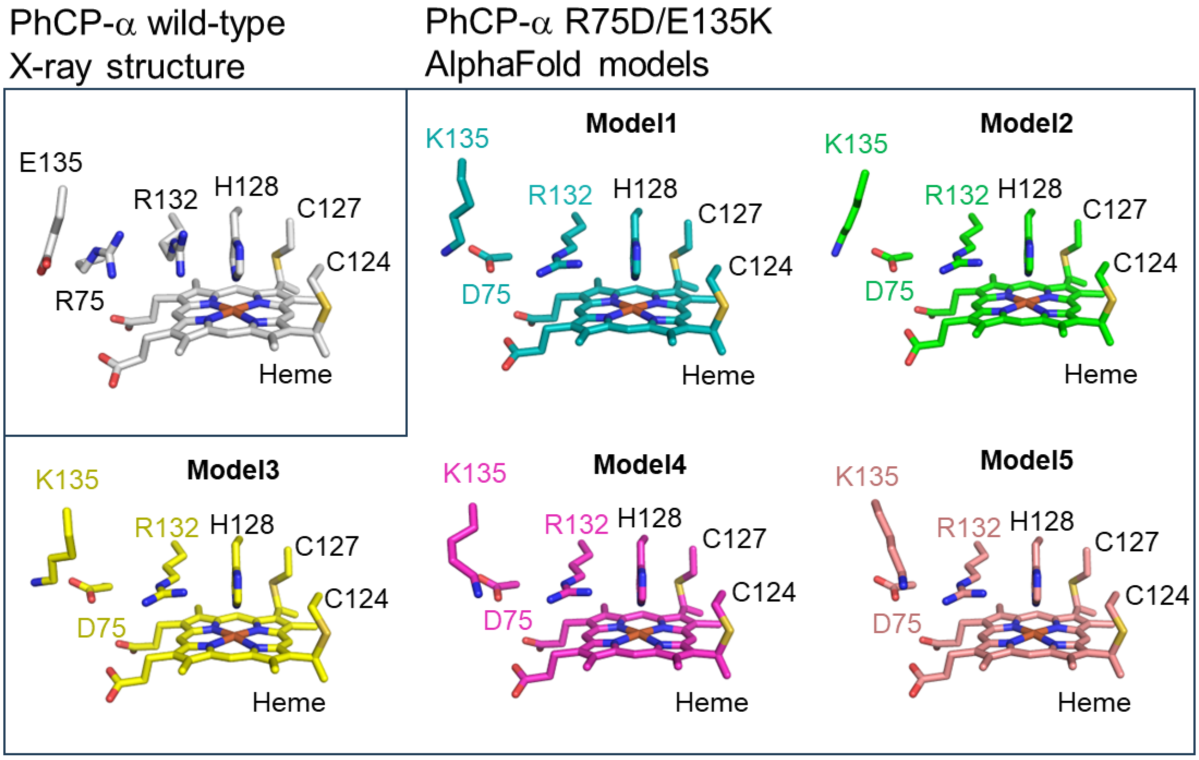

Figure S6

Heme environments of five predicted model structures in the PhCP-α R75D/E135K variant with comparing to the wild-type X-ray structure (PDB ID: 5B3I). The hemes and specific amino acid residues were drawn by sticks.
