# Supplementary material for: Conformational rigidity of cytochrome c'-α from a thermophile is associated with slow NO binding
Source: Biophys J. 2024 Jun 26;123(16):2594–603. doi: 10.1016/j.bpj.2024.06.026 (PMC11365222; doi:10.1016/j.bpj.2024.06.026)
Supplement: Document S2. Article plus supporting material [file mmc2.pdf]

Biophysical Journal
Article

Biophysical Society

Conformational rigidity of cytochrome *c*'-α from a thermophile is associated with slow NO binding

Sotaro Fujii,[1,2,3,*] Michael T. Wilson,[4] Hannah R. Adams,[4] Halina Mikolajek,[1,2] Dimitri A. Svistunenko,[4] Peter Smyth,[1,2,4] Colin R. Andrew,[5] Yoshihiro Sambongi,[3,6] and Michael A. Hough[1,2,4]

[1]Diamond Light Source Ltd, Harwell Science and Innovation Campus, Didcot, United Kingdom; [2]Research Complex at Harwell, Harwell Science and Innovation Campus, Didcot, United Kingdom; [3]Graduate School of Integrated Sciences for Life, Hiroshima University, Higashi-Hiroshima, Japan; [4]School of Life Sciences, University of Essex, Colchester, United Kingdom; [5]Department of Chemistry and Biochemistry, Eastern Oregon University, La Grande, Oregon; and [6]Seto Inland Sea Carbon-neutral Research Center, Hiroshima University, Higashi-Hiroshima, Japan

ABSTRACT Cytochromes *c*'-α are nitric oxide (NO)-binding heme proteins derived from bacteria that can thrive in a wide range of temperature environments. Studies of mesophilic *Alcaligenes xylosoxidans* cytochrome *c*'-α (AxCP-α) have revealed an unusual NO-binding mechanism involving both heme faces, in which NO first binds to form a distal hexa-coordinate Fe(II)-NO (6cNO) intermediate and then displaces the proximal His to form a proximal penta-coordinate Fe(II)-NO (5cNO) final product. Here, we characterize a thermally stable cytochrome *c*'-α from thermophilic *Hydrogenophilus thermoluteolus* (PhCP-α) to understand how protein thermal stability affects NO binding. Electron paramagnetic and resonance Raman spectroscopies reveal the formation of a PhCP-α 5cNO product, with time-resolved (stopped-flow) UV-vis absorbance indicating the involvement of a 6cNO intermediate. Relative to AxCP-α, the rates of 6cNO and 5cNO formation in PhCP-α are ~11- and ~13-fold lower, respectively. Notably, x-ray crystal structures of PhCP-α in the presence and absence of NO suggest that the sluggish formation of the proximal 5cNO product results from conformational rigidity: the Arg-132 residue (adjacent to the proximal His ligand) is held in place by a salt bridge between Arg-75 and Glu-135 (an interaction not present in AxCP-α or a psychrophilic counterpart). Overall, our data provide fresh insights into structural factors controlling NO binding in heme proteins, including 5cNO complexes relevant to eukaryotic NO sensors.

SIGNIFICANCE Nitric oxide (NO) functions as a ubiquitous biological signaling molecule, as well as a cytotoxic species at high concentrations. Iron-containing heme proteins play important and varied roles in NO sensing and detoxification, prompting research to understand how protein structure regulates this reactivity. Cytochrome *c*' is an NO-binding heme protein that is widely distributed from cold-loving (psychrophilic) to hot-loving (thermophilic) Gram-negative bacteria. Using structural and spectroscopic techniques, we show that the thermally stable cytochrome *c*' from *Hydrogenophilus thermoluteolus* exhibits slow heme-NO binding compared with mesophilic and psychrophilic counterparts, a difference that we attribute to conformational rigidity around the heme. Overall, this study deepens our understanding of the structural determinants for heme-NO binding, including those relevant to NO sensor proteins and biomimetics.

INTRODUCTION

Cytochromes *c*' are soluble periplasmic heme proteins widely distributed among Gram-negative bacteria from psychrophiles to thermophiles (1–4). The vast majority of cytochromes *c*' consist of homodimeric subunits with each monomer forming either a four-α helix bundle or a β sheet structure, called cytochrome *c*'-α or cytochrome *c*'-β, respectively (4–9). Both types of cytochromes *c*' contain a penta-coordinate heme per subunit, in which the heme Fe binds to an endogenous conserved proximal His ligand and their distal sites are vacant (5,8,10). Protein thermal stabilities of cytochromes *c*'-α exhibit a clear correlation with the optimal growth temperatures of source bacteria (3,11,12), and cytochromes *c*'-β also appear to exhibit a similar tendency (6). Therefore, cytochromes *c*'-α and *c*'-β have adequately evolved to adapt to the various temperature environments which the source bacteria inhabit. The stability differences are due to amino acid residues involving heme-related and subunit-subunit interactions (6,12,13).

Submitted March 4, 2024, and accepted for publication June 25, 2024.
*Correspondence: sofuji@hiroshima-u.ac.jp
Editor: Ronald Koder.
https://doi.org/10.1016/j.bpj.2024.06.026
© 2024 Biophysical Society. Published by Elsevier Inc.
This is an open access article under the CC BY license (http://creativecommons.org/licenses/by/4.0/).

Cytochrome *c*' has an ability to bind diatomic gases such as nitric oxide (NO) through the heme in vitro (14–17), indicating possible cellular functions as NO sensor or scavenger (18,19). NO-binding properties of cytochromes *c*' have been extensively studied in vitro using mesophilic *Alcaligenes xylosoxidans* cytochrome *c*'-α (AxCP-α) and psychrophilic *Shewanella frigidimarina* cytochrome c'-α (SfCP-α) (16,17,20,21). Stopped-flow kinetic analysis for AxCP-α reveals a stepwise NO-binding scheme: NO first binds at the vacant distal site of heme to form an observable hexa-coordinate Fe(II)-NO (6cNO) intermediate, followed by a second NO-dependent phase to form an observable penta-coordinate Fe(II)-NO (5cNO) final product on the opposite (proximal) heme face (14,22,23). In SfCP-α, only the 5cNO product is observable by stopped-flow analysis, in which a 6cNO intermediate is not detectable, possibly because of the rapid formation of 5cNO product via a putative 6cNO intermediate (17).

AxCP-α and SfCP-α are the only two wild-type cytochrome *c*'-α proteins to be structurally characterized as NO-bound forms to date. The x-ray crystal structures of NO-soaked Fe(II) AxCP-α and SfCP-α indicate that both form proximal 5cNO final products, in which the endogenous His ligand to heme is disrupted (17,23). A key residue for the 5cNO product formation from 6cNO intermediate in AxCP-α is proximal Arg-124 (23), whose side chain is considerably displaced upon NO binding, possibly accompanied with conformational rearrangement for the 5cNO product formation. The corresponding residue to Arg-124 in AxCP-α is Lys-126 in SfCP-α.

Genome analysis of thermophilic *Hydrogenophilus thermoluteolus* (formerly *Pseudomonas hydrogenothermophila*) reveals the presence of genes for nitrite and NO reduction enzymes together with a thermally stable cytochrome *c*'-α, PhCP-α (24–26), implying that PhCP-α may function as a cellular NO sensing and scavenging protein. The sequence identity of PhCP-α with AxCP-α and SfCP-α was 32 and 27%, respectively (Fig. S1 *A*). The x-ray crystal structure of native PhCP-α in the absence of NO revealed a four-α helix bundle fold, with main-chain root mean-square deviation values of 1.14 Å for AxCP-α and 1.04 Å for SfCP-α. Like AxCP-α and SfCP-α, PhCP-α contains a penta-coordinate heme in each subunit of the homodimer (Fig. S1 *B*) (12), indicating the functional conservation regarding in vitro NO binding. In this study, we characterized the NO-binding properties of PhCP-α through spectroscopic, kinetic, and crystallographic analysis, with the results obtained compared with those of AxCP-α and SfCP-α.

MATERIALS AND METHODS

Protein preparation

PhCP-α protein was heterologously expressed in *Escherichia coli* and purified as described previously (12). AxCP-α and SfCP-α proteins were prepared as described previously (17,27). The protein purity was checked by sodium dodecyl sulfate-polyacrylamide gel electrophoresis.

UV-vis absorption spectra

The PhCP-α protein (∼6 μM) dissolved in 100 mM potassium phosphate buffer (pH 7.0) was bubbled with nitrogen gas at 20°C. The Fe(II) PhCP-α solution was prepared by adding 1 mM of ascorbic acid. The Fe(II) PhCP-α product reacted with NO was then prepared by adding solution of 0.5 mM NO donor, disodium 1-(hydroxyl-NMO-azoxy)-L-proline (ProliNONOate) (Cayman Chemicals, USA) that was dissolved with a 25 mM NaOH solution (pH 11.0). One mole of ProliNONOate produces two moles of NO gas, and the NO concentration in the reaction solution was estimated from the concentration of ProliNONOate dissolved. The UV-vis absorption spectra from 350 to 700 nm were measured using a V-730BIO spectrophotometer (JASCO, Japan) at room temperature. The extinction coefficient spectra of the Fe(II) state and 5cNO product of PhCP-α were obtained using the pyridine hemochrome method to determine the protein concentration (28).

Electron paramagnetic resonance spectra

The continuous wave electron paramagnetic resonance (EPR) spectra of Fe(II) PhCP-α and its reaction product with NO were measured on a Bruker X-band E500 EPR spectrometer (Bruker, USA). A solution of 30 μM PhCP-α protein sample dissolved with 10 mM Tris-HCl buffer (pH 7.5) was prepared. The PhCP-α protein was reduced by the addition of a grain of sodium dithionite, and then 0.7 mM ProliNONOate (NO donor) was added to the resulting Fe(II) PhCP-α solution to form a NO reaction product. Syringes, needles, and EPR tubes were flushed with nitrogen before use. Immediately after the NO donor addition, 200 μL aliquots were drawn into EPR sample tubes and flash-frozen in methanol kept on dry ice. Once frozen, the samples were transferred to liquid nitrogen and stored until use. Experimental conditions were as follows: microwave frequency 9.3541 GHz, microwave power 0.63 mW, modulation amplitude 5 G, sampling time 0.025 s (800 G field range, 2048 data points), number of scans per spectrum = 1.

Resonance Raman spectra

Resonance Raman (RR) measurements were carried out on solutions of 300–500 μM Fe(II) PhCP-α and its reaction products with NO in 50 mM HEPES buffer (pH 8.0). The Fe(II) PhCP-α protein was prepared by reducing Fe(III) PhCP-α with a grain of sodium dithionite under anaerobic conditions. The reaction products with NO were prepared by reacting Fe(II) PhCP-α with (14) NO or (15) NO gas inside a septum-sealed anaerobic microcentrifuge tube. RR samples, contained in septum-sealed glass capillaries, were measured at room temperature and/or 100 K using 407 or 442 nm laser excitation (10–30 mW at the sample) for periods of 1–3 min. RR spectra were recorded on a custom McPherson 2061/207 spectrograph (McPherson, Australia) equipped with a 2400 grooves mm^{-1} holographic grating and a Princeton Instruments liquid N_2-cooled (LN-1100PB) CCD detector. An indene standard was used to calibrate Raman shifts to an accuracy of ± 1 cm^{-1}.

Kinetic measurement by stopped-flow

Solutions of 5.0 μM Fe(III) PhCP-α were deoxygenated under vacuum and placed under argon gas. Reduction of the protein was achieved by an addition of a grain of sodium L-ascorbate. The absorbance change upon NO binding to Fe(II) PhCP-α were monitored with an Applied Photophysics SX-20 stopped-flow spectrometer at 20°C. One syringe of the stopped-flow apparatus was filled with 5.0 μM deoxygenated Fe(II) PhCP-α protein

dissolved in 10 mM Tris-HCl buffer (pH 7.5), and the other was filled with 10 mM deoxygenated Tris-HCl buffer (pH 7.5) containing 1.0 mM ProliNONOate solution. Solutions of ProliNONOate at concentrations of 0.0125–0.5 mM (0.035–1.0 mM NO) were made by dilution of the stock solution into the deoxygenated buffer. The final concentration of PhCP-α was 2.5 μM for each measurement. The absorbance spectral transition in the range of 300–600 nm was monitored for 0.003–5.5 s using white light with a photodiode array detection. The global analysis of all spectral transitions was performed by Pro-K software (Applied Photophysics, UK) to fit the data into three spectral species for Fe(II) PhCP-α, 6cNO intermediate, and 5cNO final product. The stopped-flow data were further obtained with monochromatic light and a photomultiplier detector to avoid Fe-NO photolysis by white light. Under the same buffer conditions, the absorption changes at 426, 413, and 383 nm, respectively, representing Fe(II) PhCP-α, 6cNO intermediate, and 5cNO final product, were monitored for 0.003–10 s. The time course of the absorbance change was then subjected to a least-squares fitting by Pro-K software (Applied Photophysics, UK) to yield the pseudo-first-order rate constant, $k_{obs(A)}$ for the 6cNO formation and $k_{obs(B)}$ for the 5cNO formation, at each NO concentration.

Crystallization

The Fe(III) PhCP-α protein was dialyzed with milliQ water and concentrated to ~5 mg mL^{-1}. Equal amounts of protein solution and 0.1 M sodium acetate buffer (pH 4.5), 0.2 M lithium sulfate, and 30% (w/v) PEG 8000 were mixed and then equilibrated over a well containing 0.1 M sodium acetate buffer (pH 4.5), 0.2 M lithium sulfate, and 30% (w/v) PEG 8000 in a 96-well In-Situ-1 crystallization plate (MiTeGen, USA) at 20°C in a sitting drop vapor diffusion. To prepare Fe(II) PhCP-α crystals, 0.1 μL ascorbic acid (300 mM) was added to a 0.2 μL PhCP-α drop solution, and kept for 1 min under aerobic conditions. To prepare NO-soaked Fe(II) PhCP-α crystals, 0.1 μL ProliNONOate (4.5 mM) was injected into the reservoir solution containing the crystals, and incubated for 1 min. The crystals were directly flash-cooled in liquid nitrogen without cryoprotectant solution.

X-ray crystallography

X-ray diffraction data for the NO-soaked Fe(II) PhCP-α crystals were collected at beamline I04 at Diamond Light Source (Didcot, UK) using an x-ray wavelength of 0.98 Å. Data were processed with xia2.dials (29), and the resulting structure was solved by molecular replacement in Phaser (30) with a monomer of the 1.89 Å resolution crystal structure of native Fe(III) PhCP-α without NO (PDB: 5B3I) (12) as a template. The structure was refined by maximum likelihood methods using REFMAC5 (31) and modeling carried out within Coot (32), including addition of water molecules. Data collection and refinement statistics are shown in Table 1. Coordinates and structure factors of the NO-soaked crystal of Fe(II) PhCP-α protein (Fe(II) PhCP-α 5cNO product) were deposited in the PDB (PDB: 8RKP). Protein structure figures were generated with CCP4-MG and PyMOL (Schrödinger, USA). Channel structure was verified by CAVER version 3.0.1 (https://caver.cz/index.php) as a PyMOL plug-in. The statistical error values for the positions of each atom were calculated by a diffraction precision index value (http://cluster.physics.iisc.ac.in/dpi/) with errors in bond lengths derived from the diffraction precision index values of the two participating atoms (33).

Thermal stability measurement by circular dichroism

The thermal stability of the native Fe(III) AxCP-α and SfCP-α proteins (20 μM) without NO in 20 mM potassium phosphate buffer (pH 7.0) was measured by circular dichroism (CD) analysis in a pressure-proof cell compartment attached to a JASCO J-820 CD spectrometer (JASCO), as described previously (12). The temperature-dependent CD ellipticity changes at 222 nm were monitored in a cuvette of 1-mm path length at a heating rate of 1.0°C min^{-1} and at 0.9 MPa. These conditions are the same as those applied for the stability measurement of native Fe(III) PhCP-α (12). The CD ellipticity changed at 222 nm of AxCP-α and SfCP-α proteins were recorded from 30 to 100°C at intervals of 0.5°C. The recorded CD values at 222 nm were then subjected to nonlinear least-squares fitting of the thermal denaturation profile (34). The data points were corrected for the slopes of the baselines for the native and denatured forms, and then normalized to calculate the fraction of protein denatured. The fraction denatured was plotted as a function of temperature, and the resulting thermal denaturation curves were used to determine the temperature at the midpoint of the transition (T_m), which were compared with that of native Fe(III) PhCP-α determined previously (12).

TABLE 1 Crystallographic data collection and refinement statistics for PhCP-α 5cNO product

	PhCP-α 5cNO product
Resolution range (Å)	72.46–1.63 (1.66–1.63)
Space group	P62 2 2
Unit cell (Å, °)	83.67, 83.67, 88.25, 90.00, 90.00, 120.00
Unique reflection	23,390 (1135)
Multiplicity	19.7 (20.5)
Completeness (%)	100.0 (99.9)
Mean I/sigma(I)	13.4 (0.4)
Wilson B factor (Å^2)	23.98
R-merge	0.117 (4.252)
R-pim	0.027 (0.951)
$CC_{1/2}$	1.0 (0.3)
R-work	0.203
R-free	0.224
Protein residues	135
RMSD (bonds, Å)	0.0113
RMSD (angles, °)	2.235
Ramachandran favored (%)	96.9
Average B factor (Å^2)	35.09
Coordinate file in PDB	8RKP
Molecular replacement model in PDB	5B3I

Structure prediction

A structure of PhCP-α R75D/E135K variant was predicted by AlphaFold 3 via the AlphaFold Server (https://golgi.sandbox.google.com/) on May 28, 2024 (35). The amino acid sequence of PhCP-α with R75D/E135K double substitutions was analyzed in one subunit with selecting heme *c* as the ligand molecule.

RESULTS

Spectroscopic properties of Fe(II) PhCP-α with or without NO

A variety of spectroscopic techniques were used to characterize the Fe(II) PhCP-α protein and its reaction product with NO. In the presence of excess NO (1.0 mM), the Soret absorbance peak at 425 nm of Fe(II) PhCP-α (pH 7.0) shifted to 399 nm (Fig. S2), suggesting the formation of a 5cNO product, in line with AxCP-α and SfCP-α (14,17). An EPR spectrum of the Fe(II) PhCP-α protein with 1.4 mM NO at 100 K (pH 7.5) exhibited a characteristic three-line hyperfine signal (Fig. 1), resembling previous

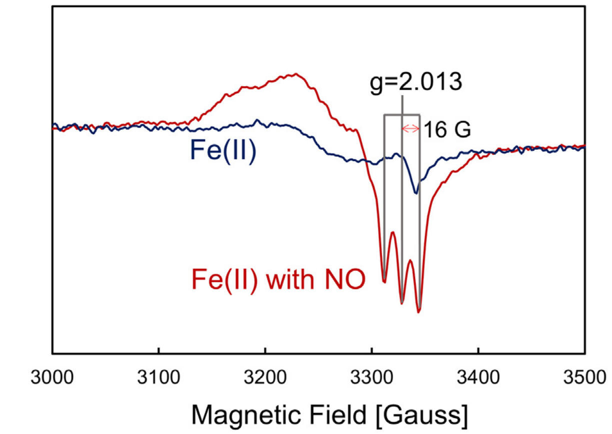

FIGURE 1 EPR spectra of Fe(II) PhCP-α with and without NO at pH 7.5 and 10 K. Red line represents the EPR spectrum of 30 μM Fe(II) PhCP-α with 1.4 mM NO, exhibiting 16 G separated three signals by hyperfine splitting around $g = 2.013$, which is indicative of the 5cNO state. Blue line represents the EPR spectrum of 30 μM Fe(II) PhCP-α without NO.

data for AxCP-α and SfCP-α (14,17), confirming that PhCP-α forms the 5cNO product with excess NO.

RR spectra of room temperature samples of Fe(II) PhCP-α, obtained with 407 or 442 nm laser excitation, featured porphyrin marker bands characteristic of the penta-coordinate high spin Fe(II) heme: ν_4 (1351 cm^{-1}), ν_3 (1471 cm^{-1}), ν_2 (1573 cm^{-1}), and ν_{10} (1606 cm^{-1}) (Fig. 2 *A*; Table 2). Cooling from room temperature to 100 K produced only minimal changes (2 cm^{-1} increases in the ν_4 and ν_3 frequencies) (Table 2). Similar to other cytochromes *c*'-α (35), Fe(II) PhCP-α exhibited a relatively high Fe-His stretching vibration (229 cm^{-1}) (Fig. 2 *A*; Table 2), indicative of a strong Fe-His bond with the imidazolate character of the proximal His ligand.

RR measurements of the Fe(II) PhCP-α 5cNO product were carried out under cryogenic conditions (100 K) to prevent loss of NO after Fe-NO photolysis with the RR laser beam. Excitation at 407 nm yielded porphyrin marker bands characteristic of a 5cNO product: ν_4 (1376 cm^{-1}), ν_3 (1508 cm^{-1}), ν_2 (1591 cm^{-1}), and ν_{10} (1641 cm^{-1}) (Fig. 2 *B*; Table 2). Substitution with NO (15) produced a -29 cm^{-1} shift in the 1661 cm^{-1} band, consistent with a ν(NO) vibration (Fig. 2 *B*; Table 2). In the low frequency region, the ν(Fe-NO) vibration was identified at 534 cm^{-1} from its 12 cm^{-1} downshift with NO (15) (Fig. 2 *B*; Table 2). Compared with cryogenic RR data for AxCP-α (Table 3), the ν(NO) frequency of PhCP-α is 3 cm^{-1} lower, and the ν(FeNO) frequency is 3 cm^{-1} higher (Table 3), suggesting that the Fe(II)→NO(π*) backbonding in PhCP-α may even be stronger than in AxCP-α (36).

Kinetics of NO binding in Fe(II) PhCP-α

Stopped-flow kinetic analysis for NO binding to the Fe(II) PhCP-α protein was first performed using white light with

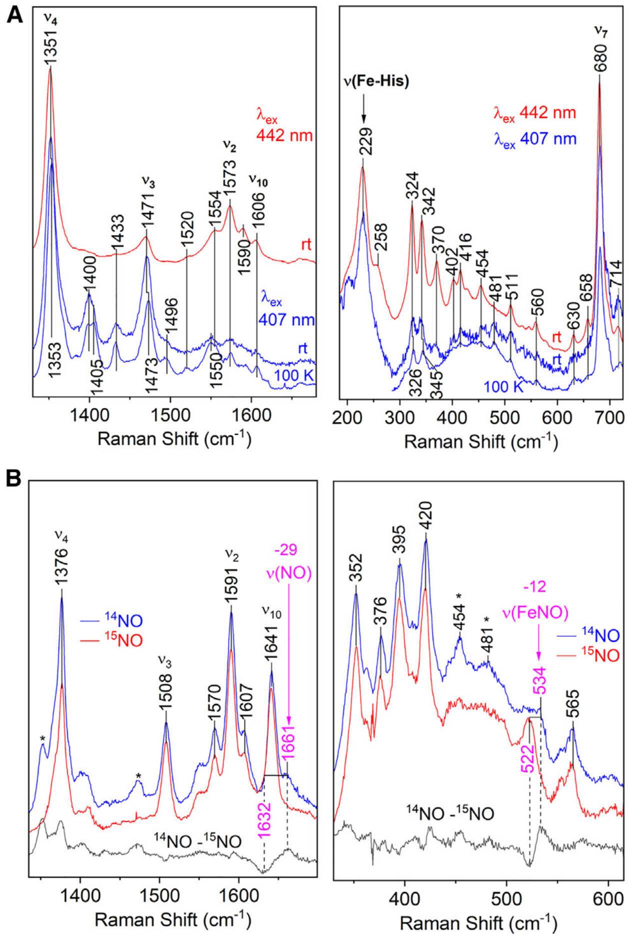

FIGURE 2 RR spectra of Fe(II) PhCP-α at pH 8.0. (*A*) Fe(II) PhCP-α (407 and 442 nm excitation) obtained at room temperature and 100 K. (*B*) 5cNO PhCP-α (407 excitation, 100 K) prepared with ^{14}NO (*blue traces*) and ^{15}NO (*red traces*). Asterisks denote contributions from a minor population of the NO-free Fe(II) state, indicating that the heme faces in the ^{14}NO sample are not fully occupied.

photodiode array detection (14,17). The spectral transitions in the range 300–600 nm were collected within 0.003–5.5 s in the presence of 0.5 mM ProliNONOate (1.0 mM NO) (Fig. 3 *A*). These were then deconvoluted into three spectral components through global analysis, exhibiting Soret peaks at 425, 416, and 399 nm (Fig. 3 *B*). The time-dependent appearance and disappearance of the Soret peak at 416 nm indicates that an intermediate 6cNO state is formed from the Fe(II) protein (Soret peak at 425 nm) before formation of a 5cNO final product (Soret peak at 399 nm) (Fig. 3 *C*), which is consistent with a biphasic kinetic model for the NO binding previously proposed in AxCP-α, yielding rate constants, k_{on} for the 6cNO formation from Fe(II) protein, and $k_{6\text{-}5}$ for 5cNO formation from the 6cNO intermediate (14).

To determine the NO-binding rates of PhCP-α, stopped-flow analysis with low NO concentration was conducted. In the presence of 0.06–0.5 mM NO, however, the absorbance amplitude values at 399 nm using the white light

TABLE 2 Characteristic RR frequencies (cm^{-1}) for Fe(II) cytochromes *c'*-α

Protein	Temperature	ν_4	ν_3	ν_2	ν_{10}	ν (Fe-His)	Reference
PhCP-α (pH 8.0)	room temperature	1351	1471	1573	1606	229	this study
	100 K	1353	1473	1573	1606		this study
AxCP-α (pH 8.9)	room temperature	1351	1469	1577	1603	231	Andrew et al. (14)

became lower than that obtained with 1.0 mM NO; for example, that with 0.06 mM NO was less than ~18% of that with 1.0 mM NO (Fig. S3 *A*). These indicate that the 5cNO product formation does not fully form under the low NO concentrations. This may be due to the Fe-NO photolysis in PhCP-α, as indicated by kinetics studies using other cytochromes *c'*-α.

To avoid Fe-NO photolysis during measurement of the NO-binding rates of PhCP-α, monochromatic light at single wavelengths, 383, 416, and 425 nm, was used for the stopped-flow analysis. By mixing 1.0 mM NO, the time course absorbance increase at 383 nm with the decrease at 426 nm was observed in PhCP-α (Fig. 3 *D*), indicating the formation of 5cNO final product from the Fe(II) protein. Increase and decrease of absorbance at 416 nm represent the appearance and disappearance of the 6cNO intermediate, respectively (Fig. 3 *D*). The fitting lines were calculated from the time-dependent absorbance changes with small residuals (Fig. S4), yielding the pseudo-first-order rate constants, $k_{obs(A)}$ for the 6cNO formation and $k_{obs(B)}$ for the 5cNO formation based on the absorbance changes at 426 nm. The k_{obs} values determined from the absorbance change at 426 nm were the same as those at 383 and 416 nm, indicating no wavelength dependence of NO binding over the range studied.

The $k_{obs(A)}$ and $k_{obs(B)}$ values of PhCP-α exhibited a linear dependency on the NO concentration from 0.0125 to 1.0 mM (Fig. 3 *E*), yielding the k_{on} value of 2.90×10^3 M^{-1} s^{-1} for the formation of 6cNO intermediate and the $k_{6\text{-}5}$ value of 4.80×10^2 M^{-1} s^{-1} for the formation of 5cNO final product. As the k_{on} value is higher than that of $k_{6\text{-}5}$ during the NO binding in PhCP-α, the formation of 5cNO is a rate-determining step, as in AxCP-α (14,22). In addition, these respective values of PhCP-α obtained at 20°C and pH 7.5 are approximately ~11- and ~13-fold lower than those of AxCP-α (3.29×10^4 M^{-1} s^{-1} for k_{on} and 6.33×10^3 M^{-1} s^{-1} for $k_{6\text{-}5}$) obtained at 20°C and pH 8.9.[22] Although there is a difference in the experimental pH conditions between PhCP-α (this study) and AxCP-α (previous study), these findings imply that the formation of 6cNO intermediate and 5cNO final product are slower in PhCP-α than in AxCP-α.

PhCP-α 5cNO crystal structure

To understand the structural reasons for the slow NO binding in PhCP-α, the crystal structure of NO-soaked Fe(II) PhCP-α was determined at 1.6 Å resolution in space group $P6_222$, which was the same as that of the native Fe(III) PhCP-α crystal without NO. The NO-soaked Fe(II) PhCP-α structure differs from that of the native PhCP-α in that the proximal His-128 ligand was dissociated from the heme center, and instead electron density consistent with an NO molecule is observed at the proximal heme face with the NO N and heme Fe distance of 1.90 ± 0.15 Å and Fe-N-O angle of 105° (Fig. 4 *A*). Thus, the NO-soaked Fe(II) PhCP-α structure represents that of proximal 5cNO product. The N-Fe distances in AxCP-α and SfCP-α (for subunit A) are 1.73 ± 0.10 and 1.75 ± 0.07 Å, respectively (Fig. 3 *B*), indicating no statistical difference in Fe-NO coordination lengths among PhCP-α, AxCP-α, and SfCP-α. The Fe-N-O angles of AxCP-α and SfCP-α (for subunit A) are at 139 and 123°, respectively, inclined in the same direction as PhCP-α.

The heme proximal region of PhCP-α 5cNO including dissociating His-128 exhibits different conformational rearrangements from those of AxCP-α and SfCP-α 5cNO products determined previously (Fig. 4 *C*) (17,23). In the PhCP-α 5cNO structure, the His-128 Cα atom moves outward from the heme center upon NO binding, and its side chain does not interact with the neighboring Glu-129 residue (Fig. 4 *C*), whereas the side chains of His ligands in AxCP-α and SfCP-α 5cNO flip at the Cβ-Cγ axis without movement of their Cα atoms and form hydrogen bonds with Asp-121 in AxCP-α and Asp-123 in SfCP-α, which correspond to the PhCP-α Glu-129 residue (Fig. 4 *C*). In particular, proximal Arg-132 in the PhCP-α 5cNO product is fixed when compared with that of the native form without NO, whereas the corresponding Arg-124 side chain in AxCP-α results in a drastic 90° rotation around the Cγ-Cδ axis before and after the NO binding (Fig. 4 *C*). Such rotation of AxCP-α Arg-124 appears to be prerequisite for the His ligand dissociation and flipping to form the 5cNO structure (23). In the SfCP-α 5cNO product, the corresponding Lys-126 residue does not move upon the NO binding

TABLE 3 RR frequencies (cm^{-1}) for 5cNO products of cytochromes *c'*-α

Protein	Temperature (K)	ν_4	ν_3	ν_2	ν_{10}	ν(FeNO)	ν(NO)	Reference
PhCP-α (pH 8.0)	100	1376	1508	1591	1641	534	1661	this study
AxCP-α (pH 7.0)	100	1375	1510	1595	1645	531	1664	Servid et al. (36)

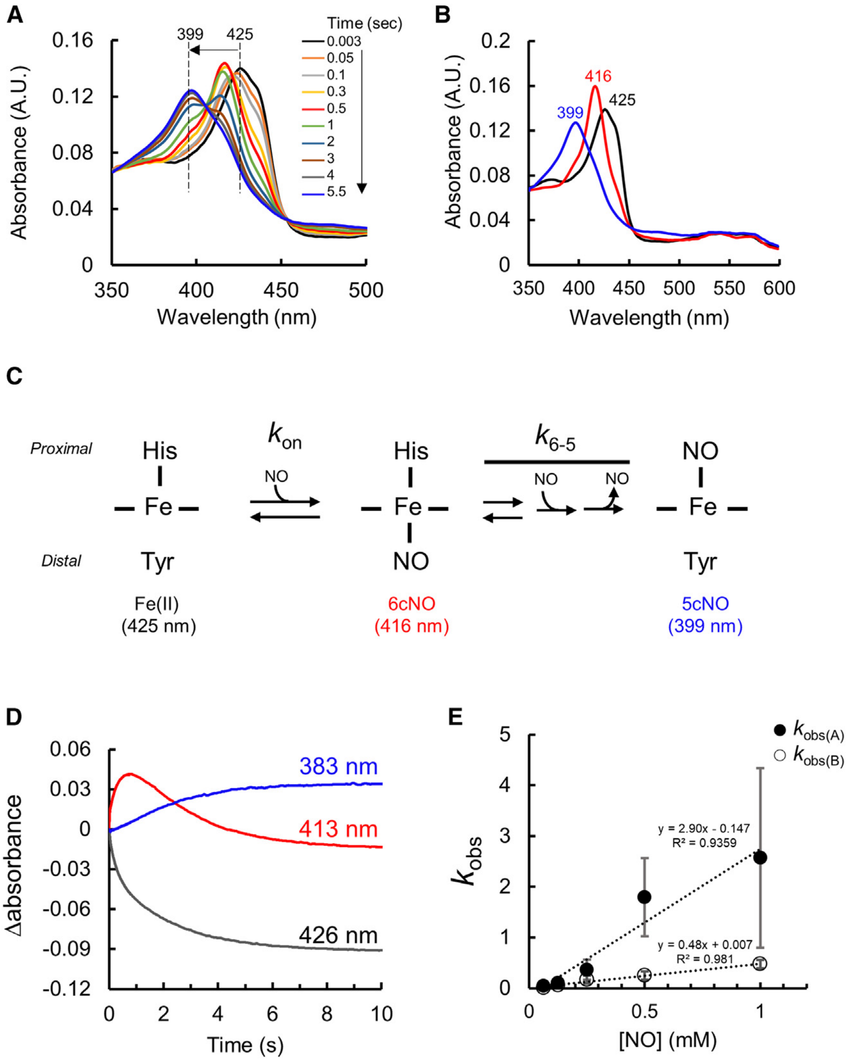

FIGURE 3 Stopped-flow kinetic analysis for PhCP-α. (*A*) Time-dependent absorbance shift of the reaction of 2.5 μM Fe(II) PhCP-α and 1.0 mM NO at pH 7.5 and 20°C by photodiode array detection using white light. (*B*) Global fitting spectra of three components of Fe(II) state (Soret peak at 425 nm), 6cNO intermediate (Soret peak at 416 nm), and 5cNO product (Soret peak at 399 nm) using the data in the presence of 1.0 mM NO. (*C*) NO-binding reaction scheme in PhCP-α based on the absorbance shift. The scheme is redrawn with the references for AxCP-α (14,23). (*D*) The representative raw data of the time-dependent absorbance shift in the reaction of 2.5 μM Fe(II) PhCP-α and 1.0 mM NO at pH 7.5 and 20°C using monochromatic light. The absorbance changes at 383, 413, and 426 nm are shown in blue, red, and gray lines, respectively. The absorbance at the start of 0.003 s is calculated as zero. (*E*) Plots of $k_{obs(A)}$ and $k_{obs(B)}$ obtained from the fitting analysis from the spectral shift at 426 nm versus NO concentration through the monochromatic light experiments. The plots indicate the means, and the error bars indicate the standard deviation based on triplicate experiments.

(Fig. 4 *C*), which may be due to the smaller side chain volume of Lys than that of Arg, enabling the His ligand dissociation and flipping in the heme proximal region of SfCP-α.

In the heme proximal region of PhCP-α, Arg-75 forms a salt bridge with Glu-135 near the Arg-132 residue before and after the NO binding (Fig. 4 *D*). This salt bridge formation may confer conformational rigidity to the heme proximal region. In contrast, the corresponding Asp-67 in AxCP-α and Glu-69 in SfCP-α do not form such salt bridges with the corresponding Lys-127 and Asp-128, respectively, in their native and 5cNO states (Fig. 4 *D*).

Thermal stability

To verify the conformational rigidity of the PhCP-α structure, thermal stability of the native AxCP-α and SfCP-α was compared with that of native PhCP-α under the same buffer conditions. The thermal stability of AxCP-α and SfCP-α was measured by CD based on the temperature-dependent signal shift at 222 nm, and then normalized (Fig. 5). The midpoint of denaturation temperature (T_m) values of AxCP-α and SfCP-α were 74.5 and 47.0°C, respectively, and both were lower than that of PhCP-α (87°C) (12), suggesting the higher conformational rigidity of PhCP-α than those of AxCP-α and SfCP-α in their native forms.

DISCUSSION

NO-binding reaction scheme in PhCP-α

In this study, we clarified the in vitro NO-binding mechanism in thermally stable PhCP-α. The NO-binding reaction scheme in PhCP-α based on the present kinetic analysis is consistent with that proposed for AxCP-α, exhibiting biphasic kinetics during the NO binding (Fig. 3 *C*) (14,23). This NO-binding mode resembles that of a eukaryotic NO sensor protein, soluble guanylate cyclase (sGC), in

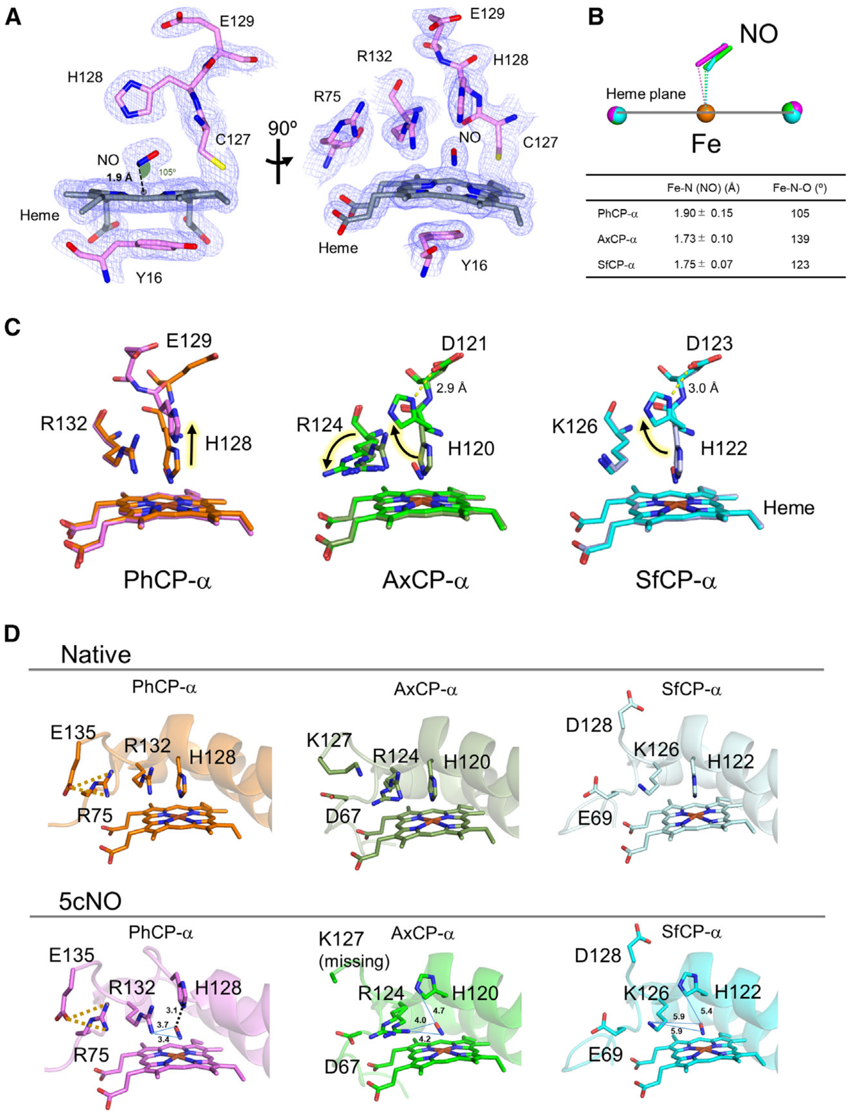

	Fe-N (NO) (Å)	Fe-N-O (°)
PhCP-α	1.90 ± 0.15	105
AxCP-α	1.73 ± 0.10	139
SfCP-α	1.75 ± 0.07	123

FIGURE 4 X-ray crystal structure comparison. (*A*) $2F_o–F_c$ electron density map of heme environment in the Fe(II) PhCP-α 5cNO product contoured at 1.5 σ. (*B*) Superposition of heme Fe and NO atoms of the 5cNO products of PhCP-α, AxCP-α, and SfCP-α. The structure colors and PDB codes used are; PhCP-α 5cNO product (*purple*, PDB: 8RKP), AxCP-α 5cNO product (*green*, PDB: 2XLM), and SfCP-α 5cNO product (*cyan*, PDB: 4CX9). The error values for each length are derived from the diffraction precision index (atomic position error values). (*C*) Superposition of the native and 5cNO states. The structure colors and PDB codes for the 5cNO products are consistent with those in (*B*), and those of native states used are; PhCP-α (*orange*, PDB: 5B3I), AxCP-α (*dark green*, PDB: 2YLI), and SfCP-α (*pale cyan*, PDB: 4ULV). (*D*) Comparison of the native and 5cNO states at the heme proximal region. The structure colors and PDB codes are consistent with those in (*B*) and (*C*). Yellow and black dotted lines indicate the salt bridge and hydrogen bond, respectively. Blue lines indicate the length of each atom with showing the distances (Å).

that two steps lead to formation of a 5cNO final product at an N-terminal heme-containing domain (19,37–39). Although cryoelectron microscopy analysis recently unveiled the activation mechanisms of sGC at 3.1–3.9 Å resolution (40,41), its x-ray crystal structure is not available to date, and its NO-binding mechanism around the heme remains unknown at the atomic level. Therefore, the 5cNO structures found in the three cytochromes *c*'-α are a valuable model for the activation mechanism in sGC at the atomic level.

Structural insights into the stronger Fe-NO backbonding in PhCP-α

RR data suggest that Fe→NO(π*) backbonding in the 5cNO product may be stronger in PhCP-α than in AxCP-α (given that PhCP-α exhibits a higher ν(Fe-NO) frequency and lower ν(NO) frequency). In the PhCP-α 5cNO structure, the Nε atom of the dissociated His-128 residue forms a hydrogen bond with the O atom of the NO ligand in the length of 3.1 Å (Fig. 4 *D*), whereas the corresponding distance from the Nε atom of His-120 to the O atom of the NO ligand in the AxCP-α 5cNO structure is 4.7 Å (Fig. 4 *D*), where it cannot form any interaction. Such hydrogen-bond formation in PhCP-α may facilitate backbonding by stabilizing the buildup of negative charge on the NO oxygen atom, akin to the trend observed for hydrogen bonding in myoglobin 6cNO product (42). It is also conceivable that the positive charge of the Arg-132 side chain could act to electrostatically enhance backbonding to the NO ligand (36). Although there is no favorable hydrogen-bonding geometry between the NO ligand and Arg-132 in PhCP-α, we note that the distance to the Nη atom (3.4 and 3.7 Å for the N and O atoms, respectively) is somewhat less

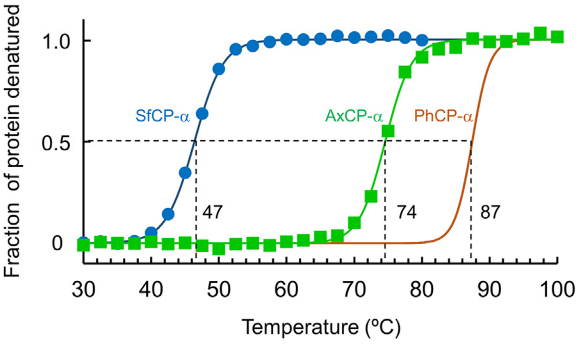

FIGURE 5 Thermal denaturation curves for native SfCP-α and AxCP-α. The normalized data for SfCP-α (*cyan circles*) and AxCP-α (*green squares*) are shown with fitting curves. The fitting curve for thermal denaturation of PhCP-α is reproduced from Fujii et al. (12). Sample conditions: protein concentration, 20 μM; solvent, 20 mM potassium phosphate buffer; pH, 7.0.

than between NO and the Nη atom of Arg-124 in AxCP-α (4.2 and 4.0 Å for the N and O atoms, respectively; Fig. 4 *D*).

Structural basis for the slow 6cNO intermediate formation in PhCP-α

The biphasic NO binding in PhCP-α yields the k_{on} value at 2.90×10^3 M^{-1} s^{-1}, which is lower than that of AxCP-α (3.29×10^4 M^{-1} s^{-1}). In native PhCP-α structure, the heme distal region is occupied by a bulky aromatic Tyr-16 residue that locates in parallel to the heme face (Fig. S1 *B*), where it can sterically hinder heme-gas coordination and/or π-stack with the porphyrin ring to possibly change the electrochemical properties (43). By contrast, the distal pocket of AxCP-α is occupied by a smaller nonaromatic residue (Leu-16), which allows the easier access of NO from the solvent to form 6cNO product. Mutations from Leu-16 to Ala and Gly in AxCP-α result in k_{on} values $\sim 10^3$–10^4 times higher (44), further supporting that a bulkier residue at the position 16 causes the slower 6cNO intermediate formation as found in PhCP-α compared with AxCP-α. In addition, PhCP-α Tyr-16 forms a hydrogen bond with Tyr-61 (Fig. S1 *B*), which may also confer the conformational rigidity at heme distal region, possibly resulting in the slower 6cNO intermediate formation in PhCP-α than in AxCP-α.

Unlike PhCP-α and AxCP-α, a 6cNO intermediate is not observable for SfCP-α by stopped-flow analysis (17). This suggests that its $k_{6\text{-}5}$ value, reflecting the rate constant from putative 6cNO to observable 5cNO product, appears to be much greater than the apparent k_{on} value of 6.89×10^3 M^{-1} s^{-1} (17). This apparent k_{on} value is close to the k_{on} value of PhCP-α, indicating that the rates for the initial NO binding to form 6cNO intermediates are likely to be similar between PhCP-α and SfCP-α. This interpretation is plausible as these two proteins similarly contain bulky aromatic residues at the distal heme face that π-stack with the porphyrin (Tyr-16 in PhCP-α and Phe-16 in SfCP-α), which may slow down the initial NO binding for the 6cNO intermediate formation compared with that in AxCP-α having Leu-16.

The solvent accessibility from the protein surface to the distal heme region also appears to affect the NO-binding affinity. However, a solvent-accessible channel to the distal heme face is only found in PhCP-α, whereas no such channel is observed in AxCP-α and SfCP-α (Fig. S5) as reported previously (12,16). Thus, rather than the presence or absence of channel structure, the local distal heme environment is likely to be the major determinant of NO binding in cytochromes *c*'-α.

Effects of salt bridge near the heme proximal region on conformational rigidity and 5cNO formation in PhCP-α

We propose that Arg-75 in PhCP-α near the heme proximal region forms a salt bridge with Glu-135 (Fig. 4 *D*), whereas the spatially corresponding Asp-67 and Lys-127 in AxCP-α do not form such interaction, suggesting that conformational rigidity near the Arg-132 residue in PhCP-α is associated with the observed slow 5cNO binding. Based on this observation, we could hypothesize that mutations of R75D/E135K on PhCP-α would convert it to an AxCP-α-like NO-binding protein. To investigate the effect of the mutations, we built a model structure of the PhCP-α R75D/E135K variant using AlphaFold 3 prediction. In the PhCP-α R75D/E135K model, the proximal Arg-132 residue was displaced toward the heme face instead of the original position (Fig. S6), which may allow the rotation of the His-128 ligand at the Cβ-Cγ axis upon NO binding, as observed in AxCP-α 5cNO structure (Fig. 4 *C*). This prediction further suggests that the salt bridge between Arg-75 and Glu-135 residues fixes the proximal Arg-132 residue, contributing to the conformational rigidity in the PhCP-α structure.

A salt bridge near the heme proximal region is also found in the x-ray structure of another thermally stable cytochrome *c*'-α from thermophilic *Thermochromatium tepidum* by the Arg-129 and Asp-131 residues (45). This salt bridge has been experimentally verified to contribute to its high thermal stability (46), implying that the PhCP-α salt bridge also contributes to the conformational rigidity around the heme. As the spectroscopic properties of *T. tepidum* cytochrome *c*'-α in the native state is similar to those of PhCP-α and AxCP-α (46,47), it will be of interest whether NO-binding rate in *T. tepidum* cytochrome *c*'-α is slow or not, which has not been examined yet.

Future experimental mutagenesis studies on PhCP-α at the distal and proximal heme region will be required to further understand the molecular mechanism of NO binding in depth. However, the present findings, obtained through the spectroscopic, kinetic, crystallographic, and thermal stability analysis, clarify similarities and differences regarding the NO binding among the three homologous cytochromes

c'-α, PhCP-α, AxCP-α, and SfCP-α derived from the thermophile, mesophile, and psychrophile, respectively. These in turn provide insights into a relationship between the NO-binding rate and thermal stability of cytochrome c'-α.

SUPPORTING MATERIAL

Supporting material can be found online at https://doi.org/10.1016/j.bpj.2024.06.026.

AUTHOR CONTRIBUTIONS

Conceptualization, S.F.; methodology, S.F., M.T.W., H.R.A., H.M., D.A.S., P.S., C.R.A., and M.A.H.; writing – original draft, S.F.; writing – review & editing, M.T.W., D.A.S., C.R.A., Y.S., and M.A.H.; visualization, S.F.; project administration, S.F.; resources, M.T.W., H.M., P.S., C.R.A., and M.A.H.; supervision, Y.S. and M.A.H.; funding acquisition, Y.S., C.R.A., and M.A.H.

ACKNOWLEDGMENTS

This work was partly supported by the Japan Society for the Promotion of Science (JSPS) Overseas Research Fellowships to S.F., the National Science Foundation (MCB-1921670) to C.R.A., and JSPS KAKENHI (23H02183) to Y.S. We also thank Dr. Moënne-Loccoz (School of Medicine, Oregon Health and Science University) for the assistance with RR measurements. P.S. was supported by a joint PhD studentship from Diamond Light Source and the University of Essex. The Crystallization Facility at Harwell is supported by Diamond Light Source Ltd, the Rosalind Franklin Institute, and the Medical Research Council. The initial stage of this project was carried out at the Diamond Light Source beamline VMXi where we acknowledge the assistance of Dr. James Sandy and Dr. Juan Sanchez-Weatherby.

DECLARATION OF INTERESTS

The authors declare no competing interests.

REFERENCES

1. Ambler, R. P. 1991. Sequence variability in bacterial cytochromes *c*. *Biochim. Biophys. Acta.* 1058:42–47.
2. Inoue, H., S. Wakai, …, Y. Sambongi. 2011. Heterologous synthesis of cytochrome c' by *Escherichia coli* is not dependent on the System I cytochrome *c* biogenesis machinery. *FEBS J.* 278:2341–2348.
3. Suka, A., H. Oki, …, Y. Sambongi. 2019. Stability of cytochromes c' from psychrophilic and piezophilic *Shewanella* species: implications for complex multiple adaptation to low temperature and high hydrostatic pressure. *Extremophiles.* 23:239–248.
4. Shibata, N., S. Iba, …, N. Yasuoka. 1998. Basis for monomer stabilization in Rhodopseudomonas palustris cytochrome c' derived from the crystal structure. *J. Mol. Biol.* 284:751–760.
5. Adams, H. R., C. Krewson, …, M. A. Hough. 2019. One fold, two functions: Cytochrome P460 and cytochrome c'-β from the methanotroph *Methylococcus capsulatus* (Bath). *Chem. Sci.* 10:3031–3041.
6. Yoshimi, T., S. Fujii, …, Y. Sambongi. 2022. Crystal structure of thermally stable homodimeric cytochrome c'-β from *Thermus thermophilus*. *Acta Crystallogr. F Struct. Biol. Commun.* 78:217–225.
7. Moore, G. R. 1991. Bacterial 4-α-helical bundle cytochromes. *Biochim. Biophys. Acta.* 1058:38–41.
8. Elmore, B. O., D. J. Bergmann, …, A. B. Hooper. 2007. Cytochromes P460 and c'-beta; A new family of high-spin cytochromes *c*. *FEBS Lett.* 581:911–916.
9. Adams, H. R., D. A. Svistunenko, …, M. A. Hough. 2023. A heme pocket aromatic quadrupole modulates gas binding to cytochrome c'-β: Implications for NO sensors. *J. Biol. Chem.* 299:104742.
10. Ambler, R. P., R. G. Bartsch, …, J. Van Beeumen. 1981. Amino acid sequences of bacterial cytochromes c' and *c*-556. *Proc. Natl. Acad. Sci. USA.* 78:6854–6857.
11. Kato, Y., S. Fujii, …, Y. Sambongi. 2015. Thermal stability of cytochrome c' from mesophilic *Shewanella amazonensis*. *Biosci. Biotechnol. Biochem.* 79:1125–1129.
12. Fujii, S., H. Oki, …, Y. Sambongi. 2017. Structural and functional insights into thermally stable cytochrome c' from a thermophile. *Protein Sci.* 26:737–748.
13. Yamane-Koshizawa, D., S. Fujii, …, Y. Sambongi. 2018. Stabilization of mesophilic *Allochromatium vinosum* cytochrome c' through specific mutations modeled by a thermophilic homologue. *Biosci. Biotechnol. Biochem.* 82:304–311.
14. Andrew, C. R., S. J. George, …, R. R. Eady. 2002. Six- to five-coordinate heme-nitrosyl conversion in cytochrome c' and its relevance to guanylate cyclase. *Biochemistry.* 41:2353–2360.
15. Stevanin, T. M., J. W. B. Moir, and R. C. Read. 2005. Nitric oxide detoxification systems enhance survival of Neisseria meningitidis in human macrophages and in nasopharyngeal mucosa. *Infect. Immun.* 73:3322–3329.
16. Hough, M. A., and C. R. Andrew. 2015. Cytochromes c': Structure, reactivity and relevance to haem-based gas sensing. *Adv. Microb. Physiol.* 67:1–84.
17. Manole, A., D. Kekilli, …, M. A. Hough. 2015. Conformational control of the binding of diatomic gases to cytochrome *c*. *J. Biol. Inorg. Chem.* 20:675–686.
18. Cross, R., D. Lloyd, …, J. W. B. Moir. 2001. Enzymatic removal of nitric oxide catalyzed by cytochrome c' in *Rhodobacter capsulatus*. *J. Bacteriol.* 183:3050–3054.
19. Mayburd, A. L., and R. J. Kassner. 2002. Mechanism and biological role of nitric oxide binding to cytochrome *c*. *Biochemistry.* 41:11582–11591.
20. Andrew, C. R., K. R. Rodgers, and R. R. Eady. 2003. A novel kinetic trap for NO release from cytochrome c': A possible mechanism for NO release from activated soluble guanylate cyclase. *J. Am. Chem. Soc.* 125:9548–9549.
21. Kekilli, D., F. S. N. Dworkowski, …, M. A. Hough. 2014. Fingerprinting redox and ligand states in haemprotein crystal structures using resonance Raman spectroscopy. *Acta Crystallogr. D Biol. Crystallogr.* 70:1289–1296.
22. Pixton, D. A., C. A. Petersen, …, C. R. Andrew. 2009. Activation parameters for heme-NO binding in *Alcaligenes xylosoxidans* cytochrome c': The putative dinitrosyl intermediate forms via a dissociative mechanism. *J. Am. Chem. Soc.* 131:4846–4853.
23. Hough, M. A., S. V. Antonyuk, …, S. S. Hasnain. 2011. Distal-to-proximal NO conversion in hemoproteins: The role of the proximal pocket. *J. Mol. Biol.* 405:395–409.
24. Goto, E., T. Kodama, and Y. Minoda. 1978. Growth and taxonomy of thermophilic hydrogen bacteria. *Agric. Biol. Chem.* 42:1305–1308.
25. Hayashi, N. R., T. Ishida, …, Y. Igarashi. 1999. *Hydrogenophilus thermoluteolus* gen. nov., sp. nov., a thermophilic, facultatively chemolithoautotrophic, hydrogen-oxidizing bacterium. *Int. J. Syst. Bacteriol.* 49 Pt 2:783–786.
26. Arai, H., Y. Shomura, …, M. Ishii. 2018. Complete genome sequence of a moderately themophilic facultative chemolithoautotrophic hydrogen-oxidizing bacterium, Hydrogenophilus thermoluteolus TH-1. *Microbiol. Resour. Announc.* 7:e008577-18.
27. Nilsson, Z. N., B. L. Mandella, …, C. R. rew. 2017. Distinguishing nitro vs nitrito coordination in cytochrome c' using vibrational spectroscopy and density functional theory. *Inorg. Chem.* 56:13205–13213.

28. Berry, E. A., and B. L. Trumpower. 1987. Simultaneous determination of hemes a, b, and c from pyridine hemochrome spectra. *Anal. Biochem.* 161:1–15.

29. Winter, G. 2010. Xia2: An expert system for macromolecular crystallography data reduction. *J. Appl. Crystallogr.* 43:186–190.

30. McCoy, A. J., R. W. Grosse-Kunstleve, …, R. J. Read. 2007. Phaser crystallographic software. *J. Appl. Crystallogr.* 40:658–674.

31. Murshudov, G. N., P. Skubák, …, A. A. Vagin. 2011. REFMAC5 for the refinement of macromolecular crystal structures. *Acta Crystallogr. D Biol. Crystallogr.* 67:355–367.

32. Emsley, P., and K. Cowtan. 2004. Coot: Model-building tools for molecular graphics. *Acta Crystallogr. D Biol. Crystallogr.* 60:2126–2132.

33. Dinesh-Kumar, K. S., M. Gurusaran, …, K. Sekar. 2015. Online_DPI: a web server to calculate the diffraction precision index for a protein structure. *J. Appl. Crystallogr.* 48:939–942.

34. Uchiyama, S., J. Hasegawa, …, Y. Kobayashi. 2002. Thermodynamic characterization of variants of mesophilic cytochrome c and its thermophilic counterpart. *Protein Eng.* 15:455–462.

35. Abramson, J., J. Adler, …, J. M. Jumper. 2024. Accurate structure prediction of biomolecular interactions with AlphaFold 3. *Nature.* 630:493–500.

36. Servid, A. E., A. L. McKay, …, C. R. rew. 2015. Resonance Raman spectra of five-coordinate heme-nitrosyl cytochromes *c*′: Effect of the proximal heme-NO environment. *Biochemistry.* 54:3320–3327.

37. Silkstone, G., S. M. Kapetanaki, …, M. T. Wilson. 2010. Nitric oxide binds to the proximal heme coordination site of the ferrocytochrome *c*/cardiolipin complex: formation mechanism and dynamics. *J. Biol. Chem.* 285:19785–19792.

38. Ma, X., N. Sayed, …, F. Van Den Akker. 2007. NO and CO differentially activate soluble guanylyl cyclase via a heme pivot-bend mechanism. *EMBO J.* 26:578–588.

39. Tsai, A. L., E. Martin, …, J. S. Olson. 2012. How do heme-protein sensors exclude oxygen? Lessons learned from cytochrome *c*′, nostoc puntiforme heme nitric oxide/oxygen-binding domain, and soluble guanylyl cyclase. *Antioxid. Redox Signal.* 17:1246–1263.

40. Kang, Y., R. Liu, …, L. Chen. 2019. Structural insights into the mechanism of human soluble guanylate cyclase. *Nature.* 574:206–210.

41. Liu, R., Y. Kang, and L. Chen. 2023. NO binds to the distal site of haem in the fully activated soluble guanylate cyclase. *Nitric Oxide.* 134–135:17–22.

42. Spiro, T. G., and A. V. Soldatova. 2012. Ambidentate H-bonding of NO and O_2 in heme proteins. *J. Inorg. Biochem.* 115:204–210.

43. Weinert, E. E., C. M. Phillips-Piro, and M. A. Marletta. 2013. Porphyrin π-stacking in a heme protein scaffold tunes gas ligand affinity. *J. Inorg. Biochem.* 127:7–12.

44. Antonyuk, S. V., N. Rustage, …, S. S. Hasnain. 2011. Carbon monoxide poisoning is prevented by the energy costs of conformational changes in gas-binding haemproteins. *Proc. Natl. Acad. Sci. USA.* 108:15780–15785.

45. Hirano, Y., Y. Kimura, …, Z. Y. Wang. 2012. Structure analysis and comparative characterization of the cytochrome *c*′ and flavocytochrome *c* from thermophilic purple photosynthetic bacterium *Thermochromatium tepidum*. *Biochemistry.* 51:6556–6567.

46. Kimura, Y., S. Kasuga, …, Z. Y. Wang-Otomo. 2015. The roles of C-terminal residues on the thermal stability and local heme environment of cytochrome *c*′ from the thermophilic purple sulfur bacterium *Thermochromatium tepidum*. *Photosynth. Res.* 124:19–29.

47. Fujii, S., S. Kobayashi, …, Y. Sambongi. 2021. Thermal stability tuning without affecting gas-binding function of *Thermochromatium tepidum* cytochrome *c*. *Biosci. Biotechnol. Biochem.* 85:1846–1852.

Biophysical Journal, Volume *123*

Supplemental information

Conformational rigidity of cytochrome *c*'-α from a thermophile is associated with slow NO binding

Sotaro Fujii, Michael T. Wilson, Hannah R. Adams, Halina Mikolajek, Dimitri A. Svistunenko, Peter Smyth, Colin R. Andrew, Yoshihiro Sambongi, and Michael A. Hough

Supplementary data

Conformational rigidity of thermophilic cytochrome *c*'-α associates with slow binding of nitric oxide

Sotaro Fujii,[a,b,c]* Michael T. Wilson,[d] Hannah R. Adams,[d] Halina Mikolajek,[a,b] Dimitri A. Svistunenko,[d] Peter Smyth,[a,b,d] Colin R. Andrew,[e] Yoshihiro Sambongi,[c,f] Michael A. Hough,[a,b,d]

[a]Diamond Light Source Ltd., Harwell Science and Innovation Campus, Didcot, UK

[b]Research Complex at Harwell, Harwell Science and Innovation Campus, Didcot, UK

[c]Graduate School of Integrated Sciences for Life, Hiroshima University, Higashi-Hiroshima, Japan

[d]School of Life Sciences, University of Essex, Wivenhoe Park, Colchester, UK

[e]Department of Chemistry and Biochemistry, Eastern Oregon University, La Grande, Oregon 97850, USA

[f]Seto Inland Sea Carbon-neutral Research Center, Hiroshima University, Higashi-Hiroshima, Japan

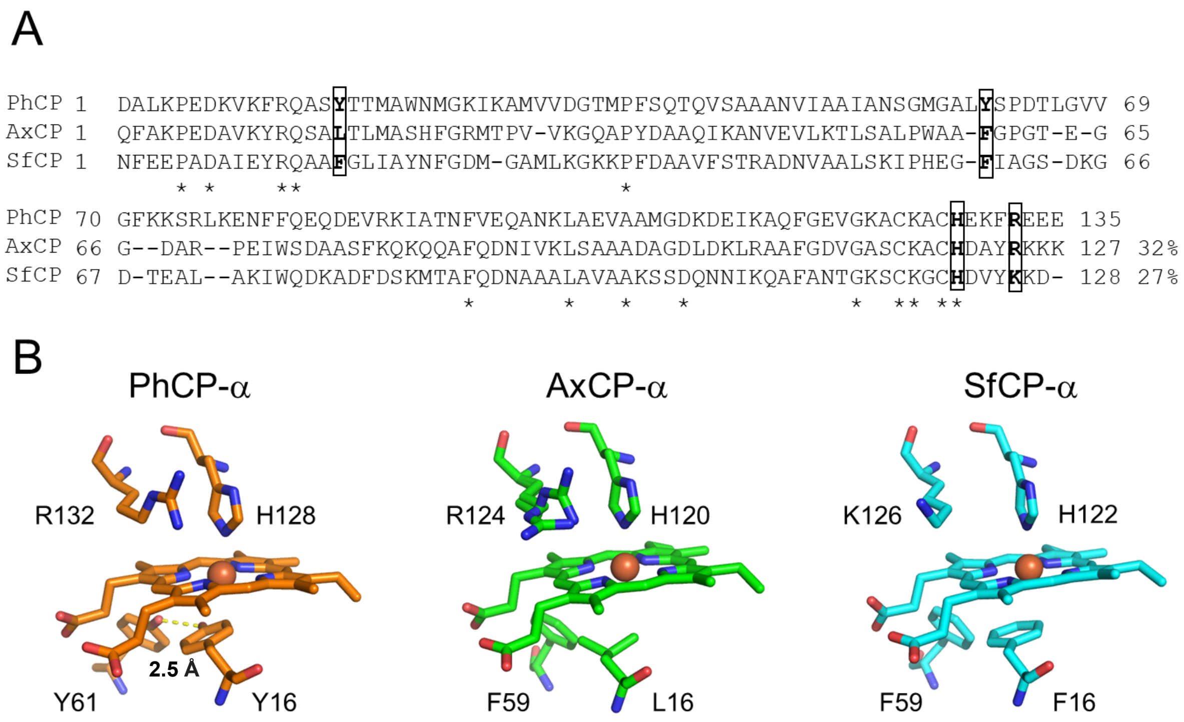

Figure S1

Structure comparison among cytochromes *c*'-α. (A) Amino acid sequence alignment of PhCP-α, AxCP-α, and SfCP-α. The relevant residues around the distal and proximal heme regions are shown in boxes. Asterisks indicate the identical residues among the three proteins. (B) Three-dimensional structure comparison of distal and proximal heme regions among PhCP-α, AxCP-α, and SfCP-α (PDB codes; 5B3I, 2YLI, and 4ULV, respectively). Boxed residues in panel A are shown. Yellow dotted line in PhCP-α indicates a hydrogen bond with a length of 2.5 Å.

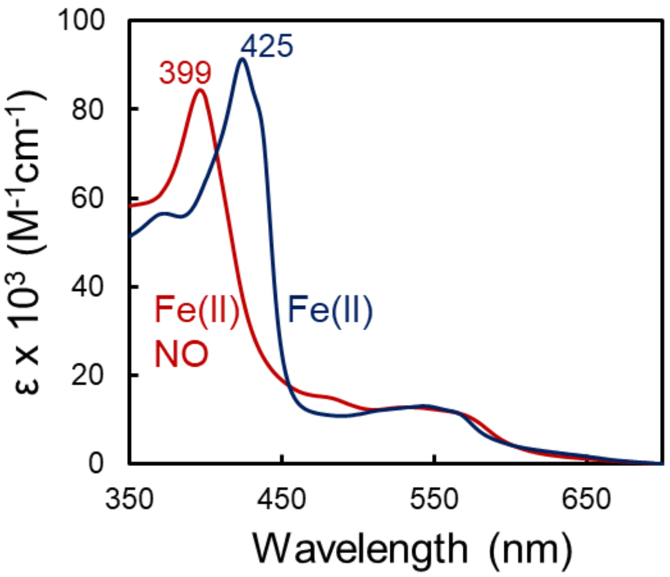

Figure S2

Molar extinction coefficient of UV-visible spectra of Fe(II) PhCP-α with NO (red line) or without NO (blue line).

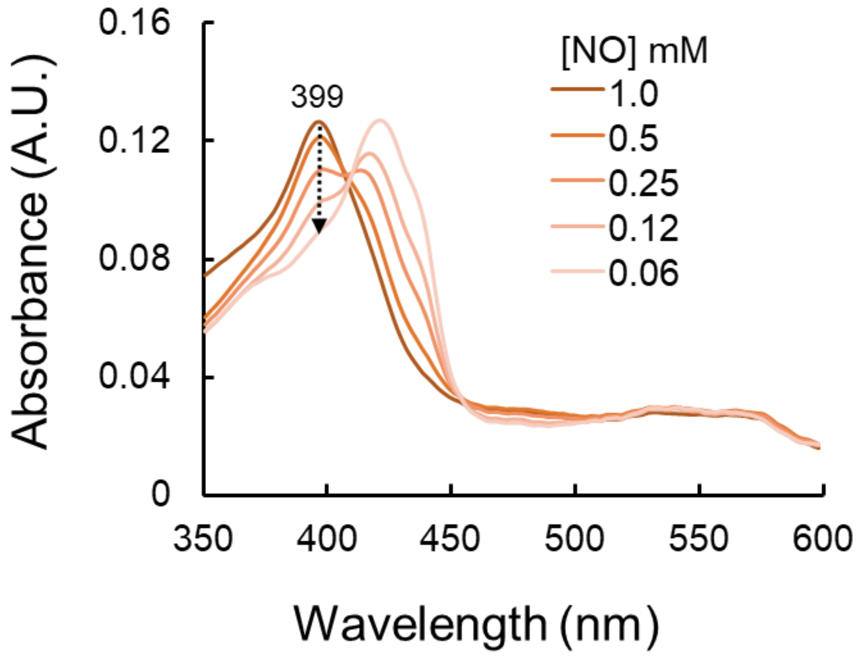

Figure S3

Absorption spectra of 2.5 µM Fe(II) PhCP-α with NO at pH 7.5 and 20 ºC after 5.5 seconds in stopped-flow analysis using white light with detecting the photodiode array.

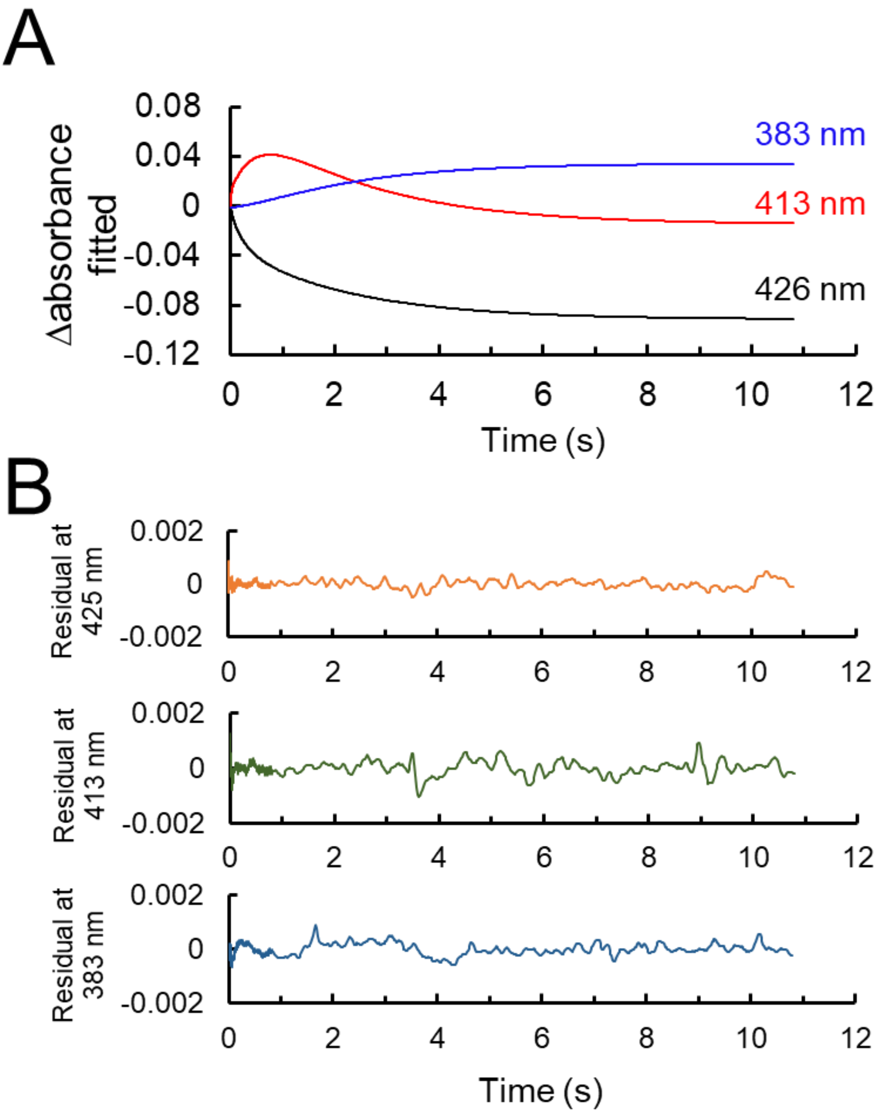

Figure S4

Stopped-flow kinetic analysis for PhCP-α using monochromatic light. (A) Fitted lines calculated from the time-dependent absorbance shift at 383 (blue line), 413 (red line), and 426 nm (black line) of the reaction of 2.5 μM Fe(II) PhCP-α and 1.0 mM NO at pH 7.5 and 20 °C. The absorbance at the start of 0.003 second is calculated as zero. (B) The residuals between raw data shown in Figure 3D and the corresponding fitted lines.

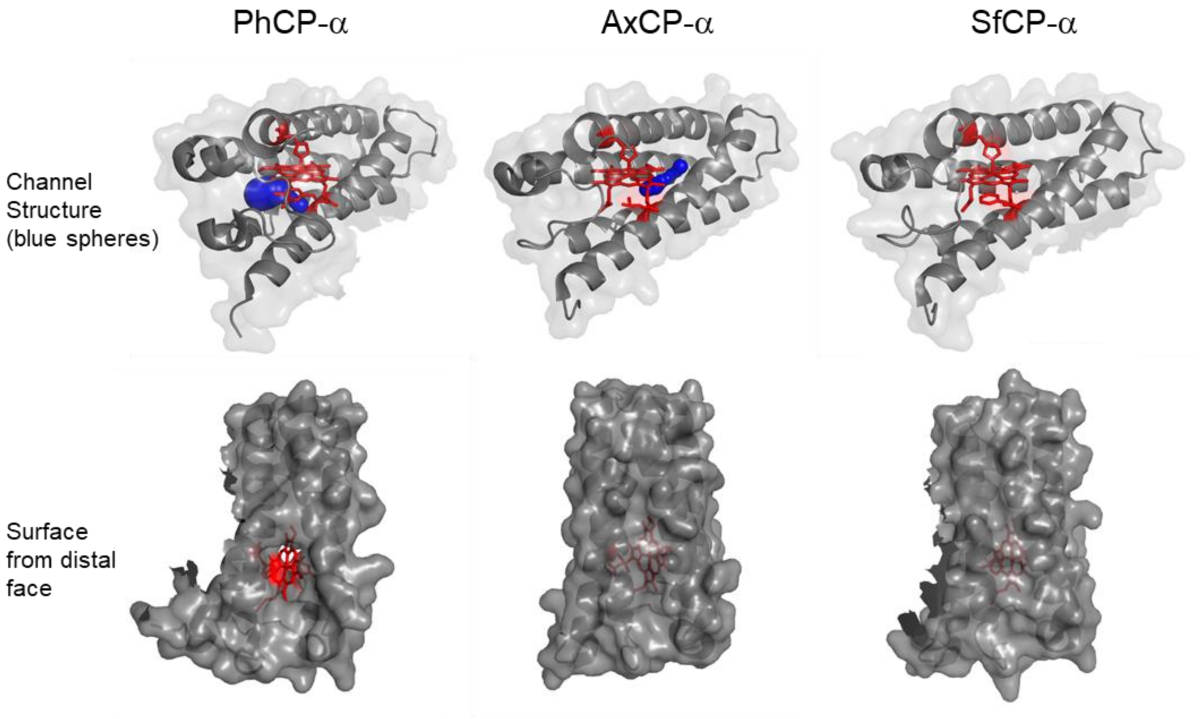

Figure S5

Channel structure analysis by CAVER program as PyMOL plugin (the top structures). The native structures of PhCP-α, AxCP-α, and SfCP-α (PDB IDs: 5B3I, 2YLI, and 4ULV, respectively) were used for the analysis. Blue spheres indicate the channel to the distal heme faces. The hemes with the coordinating distal and proximal His residues were shown by red sticks. The bottom structures indicate the protein surface shown in gray clouds, and only PhCP-α has a huge channel structure from the solvent to the distal heme face.

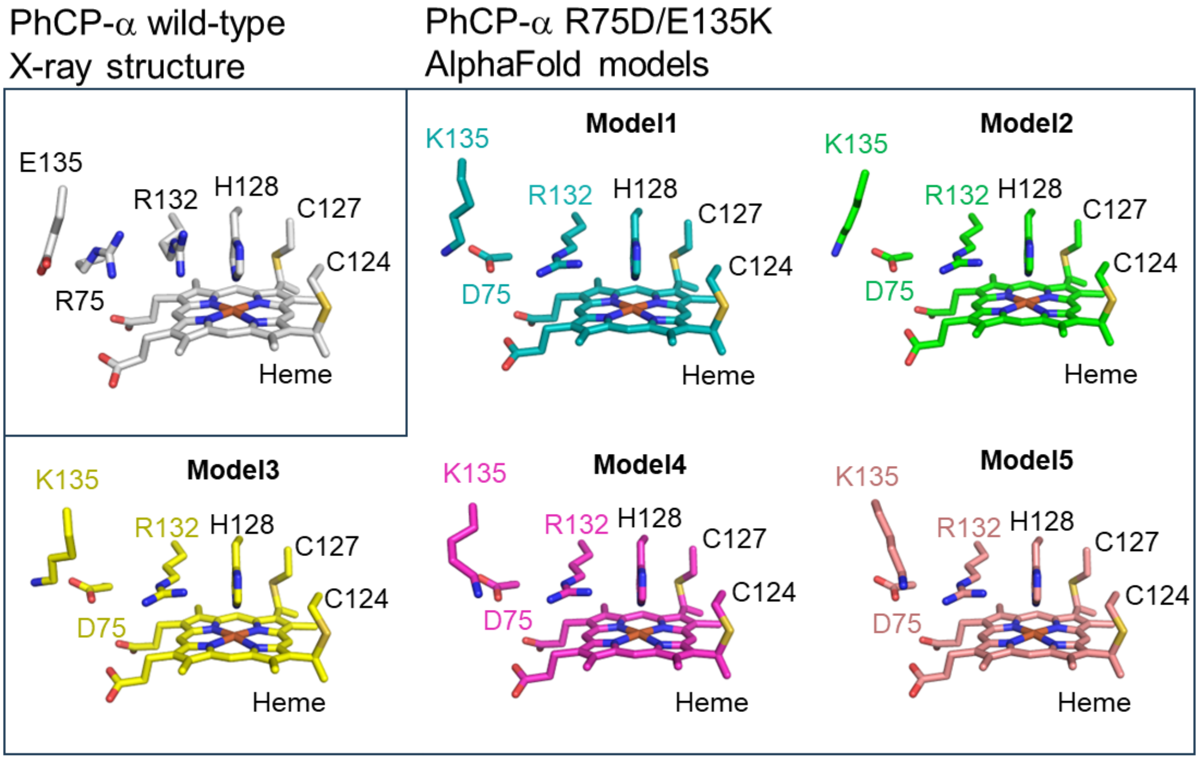

Figure S6

Heme environments of five predicted model structures in the PhCP-α R75D/E135K variant with comparing to the wild-type X-ray structure (PDB ID: 5B3I). The hemes and specific amino acid residues were drawn by sticks.
